# Supplementary material for: Shared Treg dysregulation underlies psoriasis vulgaris and atopic dermatitis through the PHF19-PRC2 epigenetic axis
Source: Front Immunol. 2026 Aug 20;17:1932788. doi: 10.3389/fimmu.2026.1932788 (PMC13538097; doi:10.3389/fimmu.2026.1932788)
Supplement: Supplementary file 3 [file DataSheet3.zip › Additional files/Additional file 4/18.RIPOR2.leaveoneout.pdf]

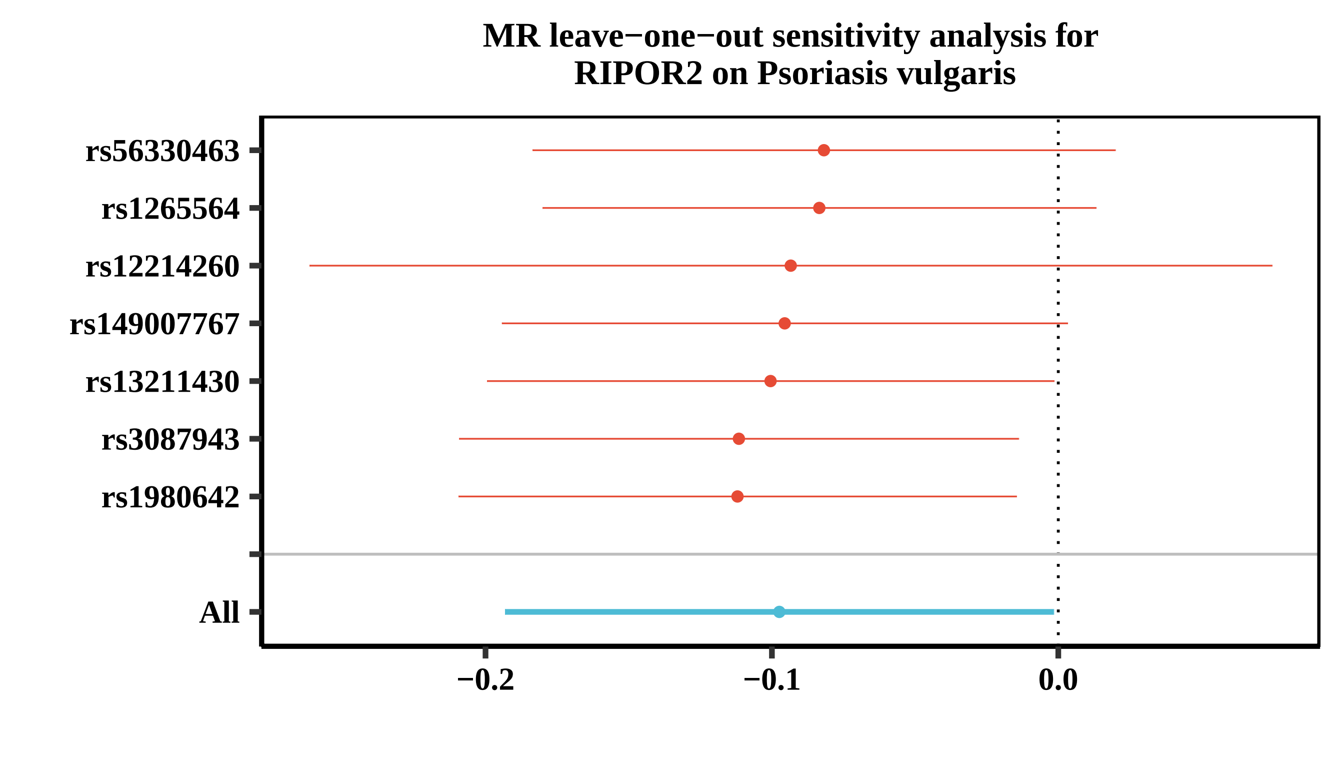

MR leave−one−out sensitivity analysis for
RIPOR2 on Psoriasis vulgaris
rs56330463
rs1265564
rs12214260
rs149007767
rs13211430
rs3087943
rs1980642
All
−0.2
−0.1
0.0
